# Numerical calculation of boundary layers and wake characteristics of high-speed trains with different lengths

**DOI:** 10.1371/journal.pone.0189798

**Published:** 2017-12-19

**Authors:** Lirong Jia, Dan Zhou, Jiqiang Niu

**Affiliations:** 1 Key Laboratory of Traffic Safety on Track, Ministry of Education, School of Traffic and Transportation Engineering, Central South University, Changsha, Hunan, China; 2 CRRC Qingdao Sifang Co. Ltd., Qingdao, China; Worcester Polytechnic Institute, UNITED STATES

## Abstract

Trains with different numbers of cars running in the open air were simulated using the delayed detached-eddy simulation (DDES). The numbers of cars included in the simulation are 3, 4, 5 and 8. The aim of this study was to investigate how train length influences the boundary layer, the wake flow, the surface pressure, the aerodynamic drag and the friction drag. To certify the accuracy of the mesh and methods, the drag coefficients from numerical simulation of trains with 3 cars were compared with those from the wind tunnel test, and agreement was obtained. The results show that the boundary layer is thicker and the wake vortices are less symmetric as the train length increases. As a result, train length greatly affects pressure. The upper surface pressure of the tail car reduced by 2.9%, the side surface pressure of the tail car reduced by 8.3% and the underneath surface pressure of the tail car reduced by 19.7% in trains that included 3 cars to those including 8 cars. In addition, train length also has a significant effect on the friction drag coefficient and the drag coefficient. The friction drag coefficient of each car in a configuration decreases along the length of the train. In a comparison between trains consisting of 3 cars to those consisting of 8 cars, the friction drag coefficient of the tail car reduced by 8.6% and the drag coefficient of the tail car reduced by 3.7%.

## Introduction

High-speed trains are a rapid form of ground transportation. With increasing speeds, the effect of a train’s aerodynamic performance is significant [1–5]. This poses a variety of questions regarding a train’s aerodynamic performance, which experts around the world have researched. While the train is running, its length changes according to the load demands. However, due to size constraints of the wind tunnel test section and the economics of the numerical calculations, we used the short train (usually 3 cars in China) to simulate the aerodynamic performance of high-speed trains in the research [6–9]. It is inaccurate to speculate the long train’s aerodynamic performance from the short train. This is because the boundary-layer thickness of the tail car, where the flow separates, varies depending on the length of the train, influencing the wake flow structures. Meanwhile, the surface pressure changes, which affect the pressure drag, result in changes to both the train’s friction drag and the aerodynamic drag. Therefore, it is necessary to analyze the flow structure and the aerodynamic performance of trains with different lengths.

There is little research regarding the flow structure of trains with differing lengths. Muld et al. [10] investigated that subject using delayed detached eddy simulations (DDES). They found that the wake flow structure had different frequencies depending on the train length, and the frequency decreased as the length increased. They attributed this to the different boundary-layer thicknesses before separation. Bell et al. [11] measured the slipstream around a high-speed train using a 1/8 scale model consisting of 2 cars in a moving model rig, and then calculated the momentum thickness using the boundary layer velocity. They found that the momentum thickness increased along the length of the train, and the side momentum thickness was sensitive to the distance above the ground. Guo et al. [12] used the improved delayed detached eddy simulation (IDDES) to study the influence of train length on the train wind. They found that bogies and bogie cabins strengthened the train wind near the ground, the train wind increased with the increasing length of the train, while the transverse train wind was not affected. There are some wind tunnel tests about the drag of trains with different lengths. Tian [13] used wind tunnel tests to conduct research regarding the drag distribution law. The result showed that the drag coefficient of the middle car depends on its location in the same configuration, and the difference could be caused by the influence of boundary layer. Huang et al. [14] also used a wind tunnel test to study the distribution regularity of different length trains, the results showed that the drag coefficient of the head and the tail car changed little with the increasing length of the train. In addition, some papers involve the comparison of the data from the numerical simulation of the short train and the full-scale test of the long train. Huang et al. [15] compared the slipstream data obtained in the numerical simulations performed with 4 cars and the full-scale test performed with 8 cars. The simulation curve, which was presented with an inserted gap equal to the length of 4 cars, compared the nose and wake flows. The researchers found that the velocity and pressure peak of the train’s tail with 4 cars were smaller than those were with 8 cars. Hemida et al. [16] also conducted a similar study. These studies focused largely on the slipstream and drag of trains with varying lengths. However, there is little research regarding the boundary layer and the flow structure around trains with different lengths and what effect they may have on the surface pressure, aerodynamic drag and friction drag.

Through the use of DDES allows for the analysis of boundary layers, wake vortices, surface pressures, friction drag coefficients and aerodynamic drag coefficients of trains with varying lengths that running in open air. The work is organized as follows. Section 2 describes the geometry, computational domains, boundary conditions and meshes around the trains, as well as the numerical details. Next, the numerical results are compared with the wind tunnel test results to check the accuracy of the mesh resolution and the method. Section 3 analyzes the boundary layers and wake vortices of trains with different lengths. Based on the flow structure, the surface pressure, friction drag coefficients and drag coefficients for trains with different lengths are also studied. Finally, conclusions are shown in Section 4.

## Numerical model

### Numerical method

The numerical simulations in this paper were conducted using Delayed Detached Eddy Simulations (DDES). DDES is considered to be a hybrid of unsteady Reynolds-Averaged Navier-Stokes (RANS) and Large Eddy Simulation (LES). Unsteady RANS was adopted in the region of the boundary layer, and cost fewer computational resources compared to the LES. LES was used in the rest of the region, and it allowed for the performance of a large scale separation structure. A SST k-ω model was chosen in RANS. More information regarding the transportation equation can be found in Menter [17]. In DDES, a model length scale, *d*_*DDES*_, is used to switch between the RANS and LES. *d*_*DDES*_ is defined as:

$$d_{DDES}=d-f_{d}\max\left(0,d-C_{DES}\Delta \right)$$ (1)

where *d* is the distance between the wall and the first cell.is the largest local grid in (Δ*x*,Δ*y*,Δ*z*), the default constant *C_DES_* in the model is 0.65.

$$f_{d}=1-\tanh\left({\left[8r_{d}\right]}^{3}\right)$$ (2)

$$r_{d}=\frac{\nu_{t}+\nu }{\sqrt{U_{ij}U_{ij}}\kappa^{2}y^{2}}$$ (3)

Here, *ν_t_* is the kinematic viscosity, *ν* is the eddy viscosity, *U_ij_* is the velocity gradient, *κ* is the Karman constant. *f_d_* is 1 in the LES region, and 0 in the RANS region [18].

### Train geometry and grid

**Fig 1:**
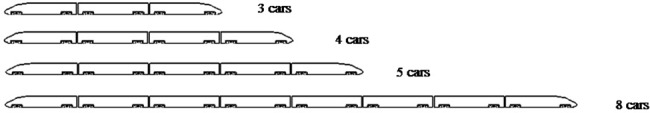
The four train configurations of the high-speed train, with 3, 4, 5, 8 cars.

**Fig 2:**
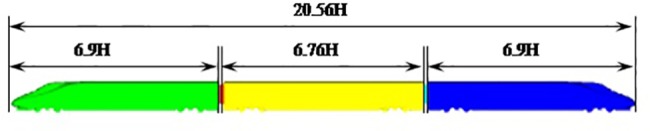
Train model of 3 cars.

The train geometry considered in this paper is a high-speed train at 1:8 scale containing bogies and inter-carriage gaps. Four different configurations with varying numbers of cars were simulated, and each configuration contained a head car, some middle cars and a tail car. According to the total number of cars, the symbols of 3 cars, 4 cars, 5 cars and 8 cars represented the 4 configurations, as shown in Fig 1. For simplicity, H denotes the height of the scaled train. So the total length (*L*) was *L = 6*.*9 H+ n* × (*6*.*76 H)+6*.*9 H*. The train model is shown in Fig 2 by the example of 3 cars.

**Fig 3:**
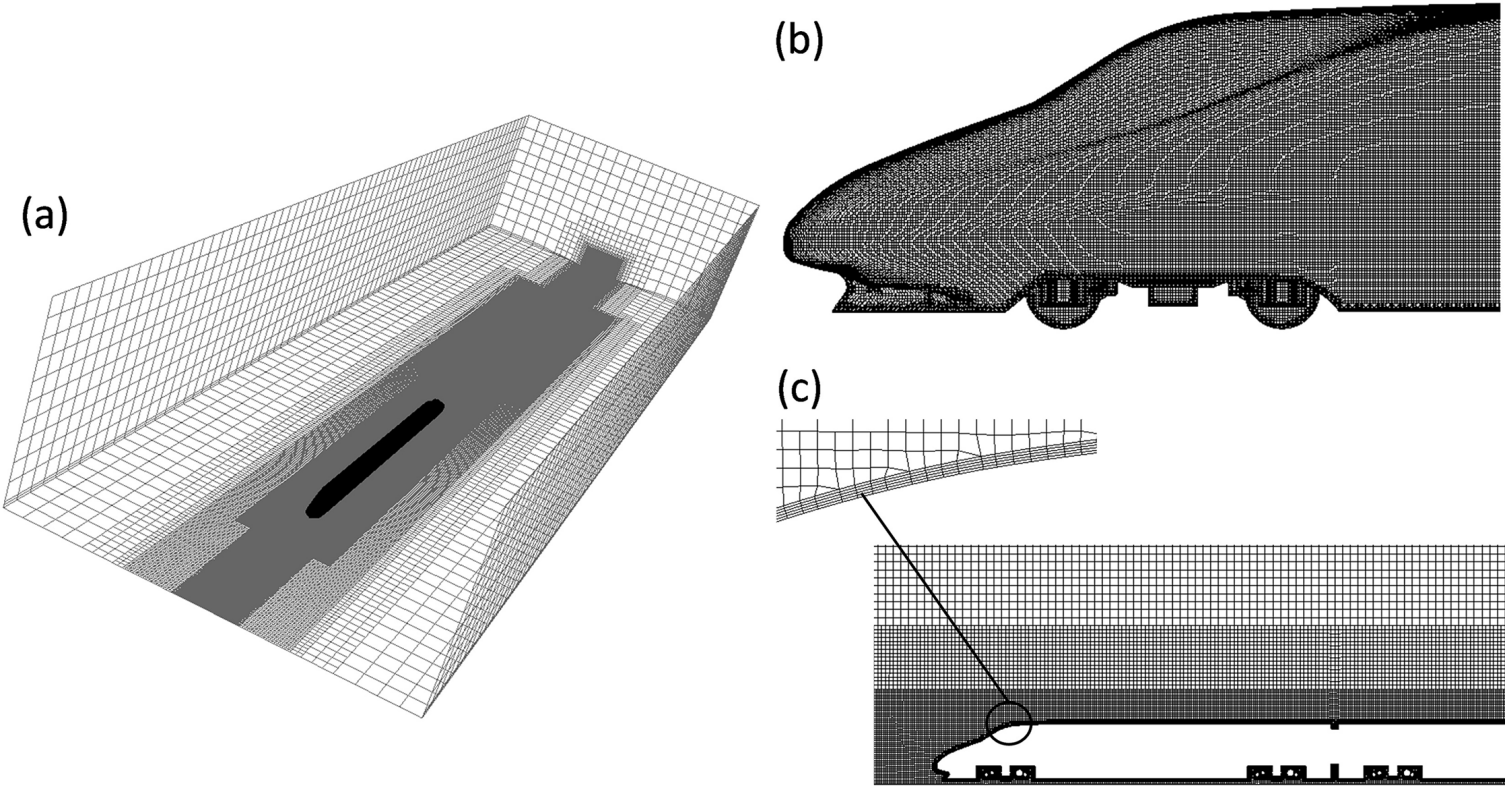
Mesh distribution around the train: (a) the whole mesh; (b) surface mesh around the head car; (c) longitudinal cut-plane mesh.

The grids of the models were generated using the SnappyHexa mesh utility which was within OpenFOAM2.3.1, The meshes were dominated by hexahedral cells. Three refinement regions were applied in the flow field around the train. Five prism layers were created around the train surface to solve the boundary layer. To guarantee the use of the k-ω turbulence model in the wall region, the first boundary-layer thickness was 0.39 mm. Based on the initial calculations, and adaptive mesh technology in Fluent was used to optimize the mesh. The mesh around the model is shown in Fig 3 (trains with 3 cars as an example).

### Computer domain and boundary conditions

**Fig 4:**
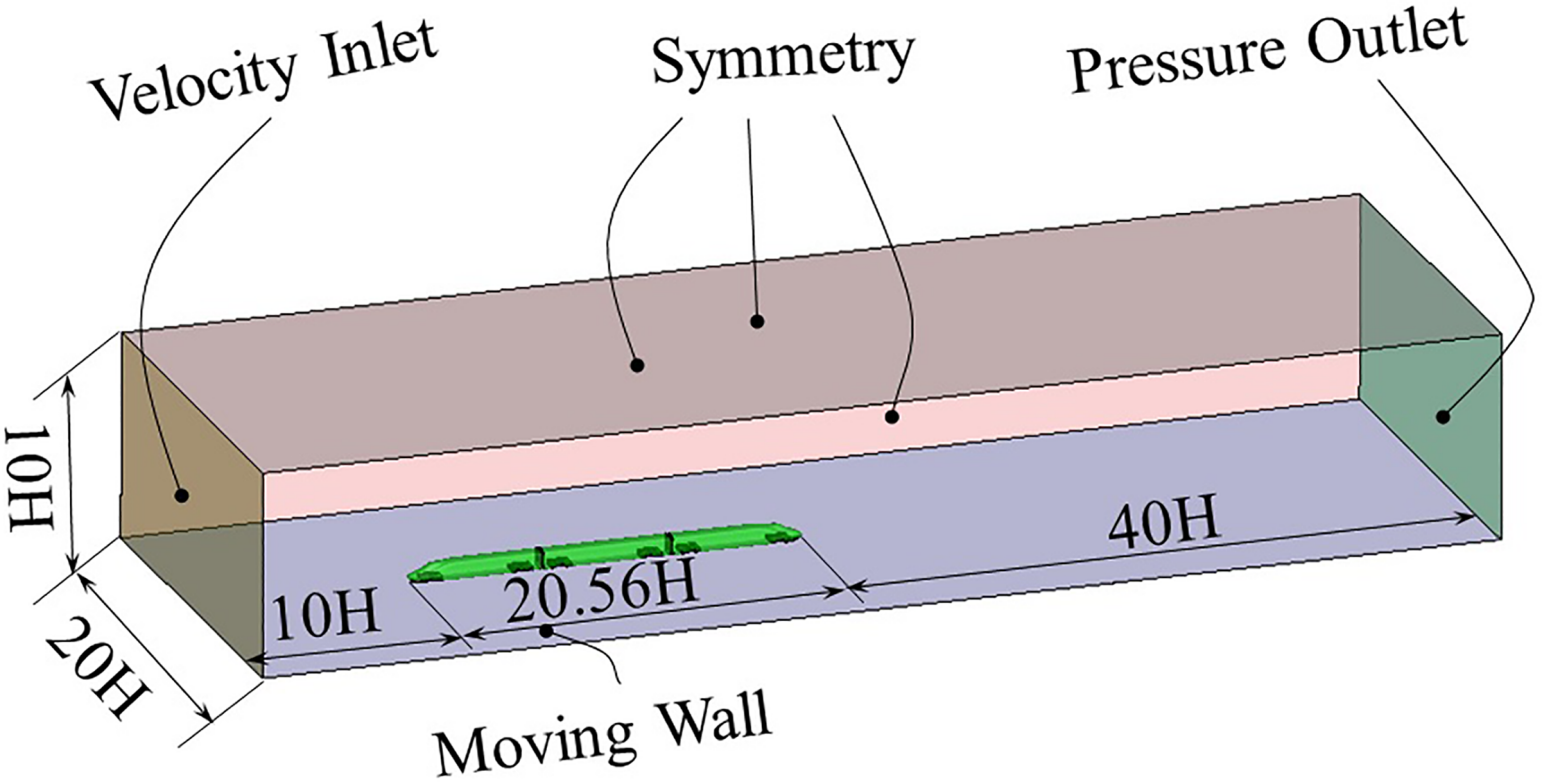
Computational domain and boundary conditions.

To guarantee the full development of the flow field and reduce the influence of boundary conditions to the flow around the train, the computer domain should be large enough. Additionally, the computer domain was similar for all 4 configurations. The distances in front and behind the train were the same. Consequently, the length of the computation domain is 70.56 H, 77.32 H, 84.08 H and 104.36 H for 3 cars, 4 cars, 5 cars and 8 cars. Also, the boundary conditions were the same. The computer domain and boundary conditions are shown in Fig 4 using the example of 3 cars. The distance before the train is 10 H, and the distance behind the train is 40 H. The height of the domain is 10H and the width is 20 H with the train model at the center of the domain. The inlet is set to the velocity inlet which is given the velocity of [*U*_*m*_, 0, 0]. Turbulent kinetic energy (κ) and special dissipation rate (ω) were calculated using Eqs (4) and (5):

$$\kappa =3/2{\left(IU_{m}\right)}^{2}$$ (4)

$$\omega =\kappa^{1/2}/\left(C_{\mu }^{1/4}*0.07H\right)$$ (5)

where *U*_*m*_ is the average air speed of the velocity inlet, which is 60 m/s. *I* is the turbulence intensity, which is 0.5% estimated from the experiment. H is the characteristic length, which is 0.4625 m. *C*_*μ*_ is the empirical constant, which is 0.09. The outlet is set to the pressure outlet, which is 0 Pa. Finally, a no-slip boundary condition was used on the train and a moving wall boundary condition was used on the ground.

### Numerical details

The Mach number is about 0.16 based on the velocity. Therefore the flow is considered to be the incompressible viscous flow. The Reynolds number is defined as:

$$R_{e}=\frac{UH}{\mu_{t}}$$ (6)

where U is the velocity, which is 60m/s, H is the train height, which is 0.4625m. Additionally, μ_t_ is the kinematic viscosity, which is 1.8 × 10^5^ m^2^/s. The Reynolds number is 1.4 × 10^6^, meaning the flow around the train is highly turbulent. The numerical simulations in this paper were conducted using the commercial solver Fluent. An unsteady pressure-based solver was used in the simulation. The Semi-Implicit Method for Pressure-Linked Equations (SIMPLE) algorithm was utilized to couple the pressure and the velocity. A standard discretization scheme was used to calculate the pressure. A bounded central difference scheme was utilized for solving the momentum equation. A second-order upwind scheme was selected for κ and ω equations. A second order implicit scheme was used to discretize the time derivative terms. The time step was 1.0 × 10^−4^ s, the inner iteration steps were 30, and the calculation could be converged after 30 iterations. A converge criterion of residuals for all flow variables was up to 10^−5^.

### Aerodynamic coefficients

For analysis, the aerodynamic coefficients were calculated as follows:

$$C_{D}=\frac{D}{0.5\rho {U_{m}}^{2}S}$$ (7)

$$C_{D\tau }=\frac{D_{\tau }}{0.5\rho {U_{m}}^{2}S}$$ (8)

$$C_{p}=\left(P-P_{0}\right)/\left(0.5\rho {U_{m}}^{2}\right)$$ (9)

Where *C*_*D*_ is the drag coefficient, and *C*_*Dτ*_ is the viscous drag coefficient. *C*_*P*_ *i*s the pressure coefficient. *ρ* is the density of air, and *U*_*m*_ is the average inlet velocity. *D* is the drag, *D*_*τ*_ is the viscous drag. *P*_*0*_ is the reference pressure, which is 0 pa. *P* is the static pressure of the surface of the train. *S* is the cross-section area of the train, which is 0.175 m^2^.

To eliminate the transient effects of the flow fields, obtaining these data began after the flow-through time passed the computational domain twice. After that, the flow-through times were run twice to obtain the time-averaged flow field, and the drag and pressure were calculated by taking the averaged value when the flow field became steady.

### Validation of the simulation

The wind tunnel test was conducted in the second test section of an 8 m × 6 m wind tunnel in China’s Aerodynamics Research and Development Center. The center’s test section is comprised of a large low-speed wind tunnel with closed tandem double test sections. A special floor consisting of 5 boards was installed in the test section. The distance between the surface of the floor and the bottom of the wind tunnel was 1.06 m. To reduce any disturbance to the flow, the floor was machined to the streamlined leading and trailing edge. After installing the floor, the effective size of the test section was 16.1 m long, 8.0 m wide and 4.9 m high. The cross-section area was 39.2 m^2^. The turbulence intensity was 0.5%, which can provide high quality inflow [19–20].

**Fig 5:**
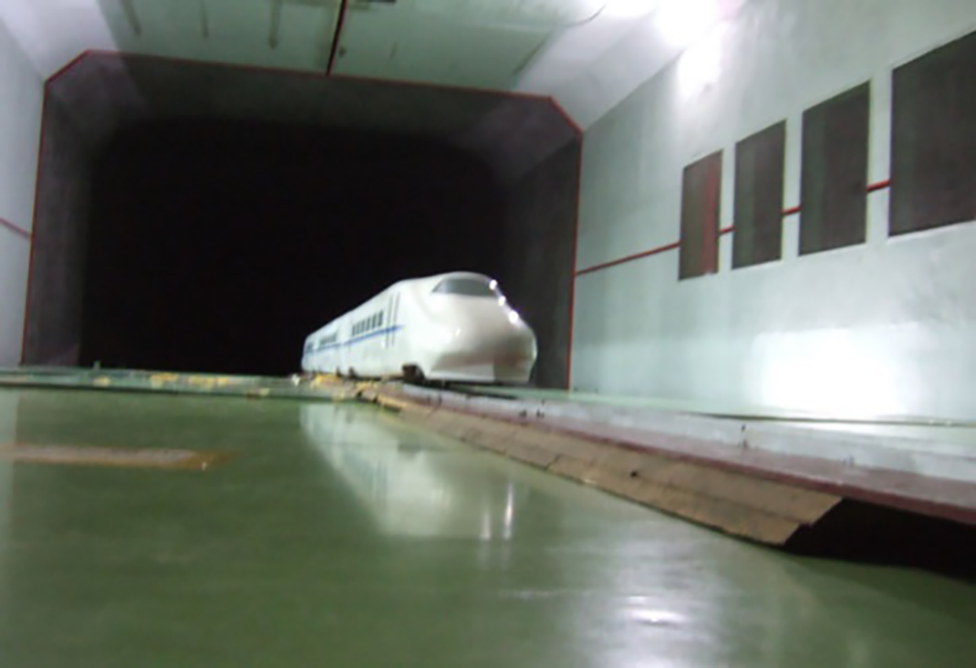
Test model.

The high-speed train test model consisted of 3 cars at 1:8 scaled, and included the roadbed and track. The track was fixed parallel to the surface of the roadbed. They were set at the bottom of the train wheels, and were 14 m long which extended about 2 m away from the head car and tail car. The front and sides of the embankment were oblique. The test model is shown in Fig 5.

A scaled numerical model identical to the wind tunnel model was established. In order to obtain the same Reynolds and Mach number as was found in the wind tunnel test, the velocity inlet was given to 60 m/s, which was used in the wind tunnel test, additional, the ground was set to the no-slip wall, which is consistent with the wind tunnel test. A mesh-independent test was performed in order to eliminate the effect of mesh density on the results. The meshes were coarse, medium and fine, consisting of 18 million, 24 million and 36 million cells, respectively. Table 1 shows the numerical results generated in different meshes. The results show a minor difference between the medium mesh and the fine mesh, which indicates that the medium mesh is refined enough and there is no need for further refinement.

**Table 1:** *C*_*D*_ from the numerical and wind tunnel test.

Method	Mesh resolution	Head car	Middle car	Tail car
Numerical simulation	coarse	0.167	0.074	0.148
	medium	0.142	0.081	0.155
	fine	0.143	0.080	0.157
Wind tunnel test		0.145	0.084	0.163

In order to check the accuracy of the method used in this paper, a comparison was made between the data obtained from numerical simulation using medium mesh and the data collected from the wind test with a maximum error of less than 5%, which certifies the method’s accuracy.

## Results

### Flow distribution around trains with different lengths

**Fig 6:**
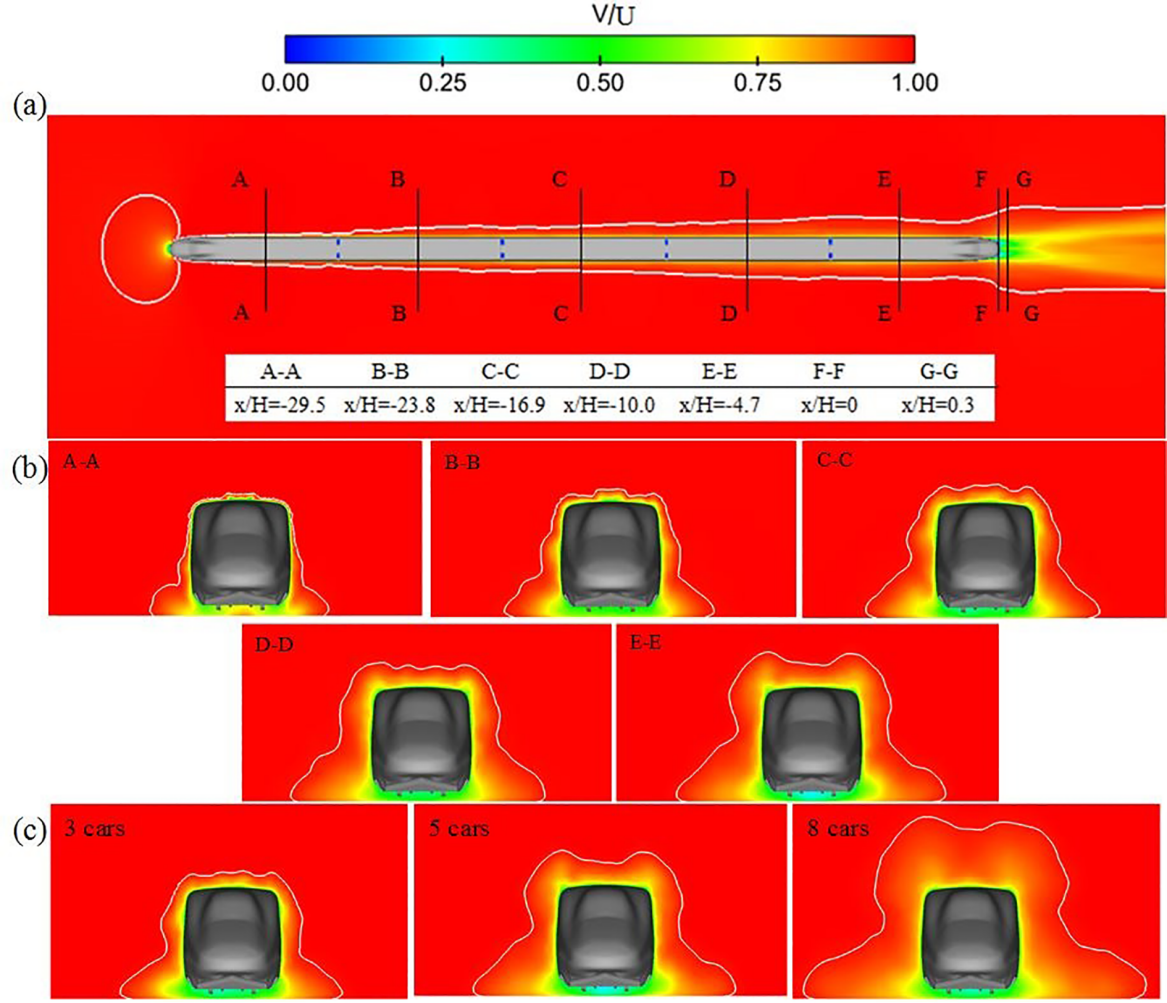
Boundary layer around the train:(a) the boundary-layer thickness on the horizontal plane in 5 cars; (b) the boundary-layer thickness of the middle section of each car in 5cars; (c) the boundary-layer thickness of the tail car for trains with 3 cars, 5 cars, 8 cars.

Fig 6(A) shows the boundary-layer thickness with a value of 99% of the inflow velocity on the horizontal plane through the nose point [21]. The figure also shows the location of cut-planes in 5 cars. Fig 6(B) shows the boundary-layer thickness of the middle section of each car(shown in Fig 6A) for 5 cars. Fig 6(C) shows the boundary-layer thickness of the middle section of the tail car for 3 cars, 5 cars and 8 cars. These figures are colored by the time-averaged velocity contour.

In Fig 6(A), the boundary-layer thickness has a small fluctuation in the near wake, but this does not affect the overall development. It can be seen that the boundary-layer thickness increases along the length of the train.

In Fig 6(B), the side boundary-layer thickness is sensitive to the distance above the ground. The boundary-layer thickness from the lower position above the ground is thicker than what is found from the higher position, which is consistent with the results from the moving model rig (Bell, 2015). This difference could be caused by the flow from the head car’s cowcatcher. The rate of the increase in the roof boundary-layer thickness is higher than that of the side boundary layer. This is because the flow is diverged from the side of the train and converges above the roof of the train (Baker, 2010). The increasing boundary-layer thickness along the length of the train is obvious.

Fig 6(C) enables a direct comparison of the boundary-layer thickness of the tail car in 3 cars, 5 cars and 8 cars, which is this paper’s focus. The boundary-layer thickness of the tail car increases with the increasing length of the train. The boundary-layer thickness is similar when comparing the tail car in 3 cars and the third car in 5 cars, further certifying that the boundary-layer thickness relates largely to the length of the train.

**Fig 7:**
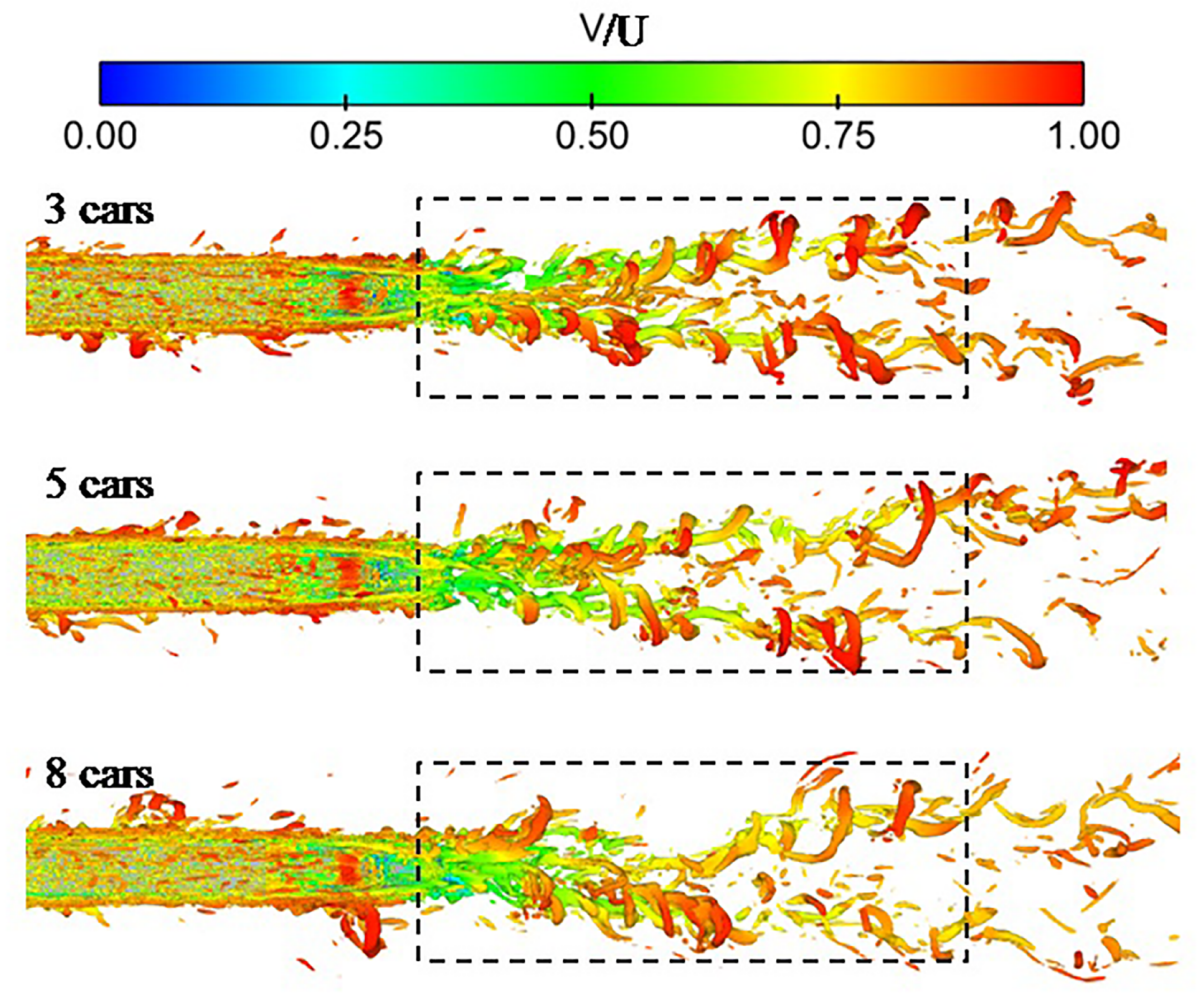
Instantaneous iso-surface plot of Q-criteria colored by mean velocity (Q = 50000).

Fig 7 shows the instantaneous vortex structure in the near wake in 3 cars, 5 cars and 8 cars, coloured by the mean velocity. The instantaneous vortex structure is depicted using the iso-surface of the second invariant of the velocity gradient(Q) [22]. Q is defined as

$$Q=-1/2\partial u_{i,j}\partial u_{j,i}$$ (10)

In Fig 7, the flow vortex structures in the near wake are similar among trains with different lengths. At the rear of the train, the attached boundary layer separates to form a pair of helical vortices extending behind the train. However, these vortices are more chaotic for a longer train, which is why the boundary-layer thickness increases with the increasing length of the train. The turbulence flow in the thicker boundary layer is more complicated, and the downwash flow makes the wake vortices more chaotic. Simultaneously, the velocity of the wake vortices decreases with the increasing length of the train, which can be attributed to the longer train consuming a greater amount of energy.

**Fig 8:**
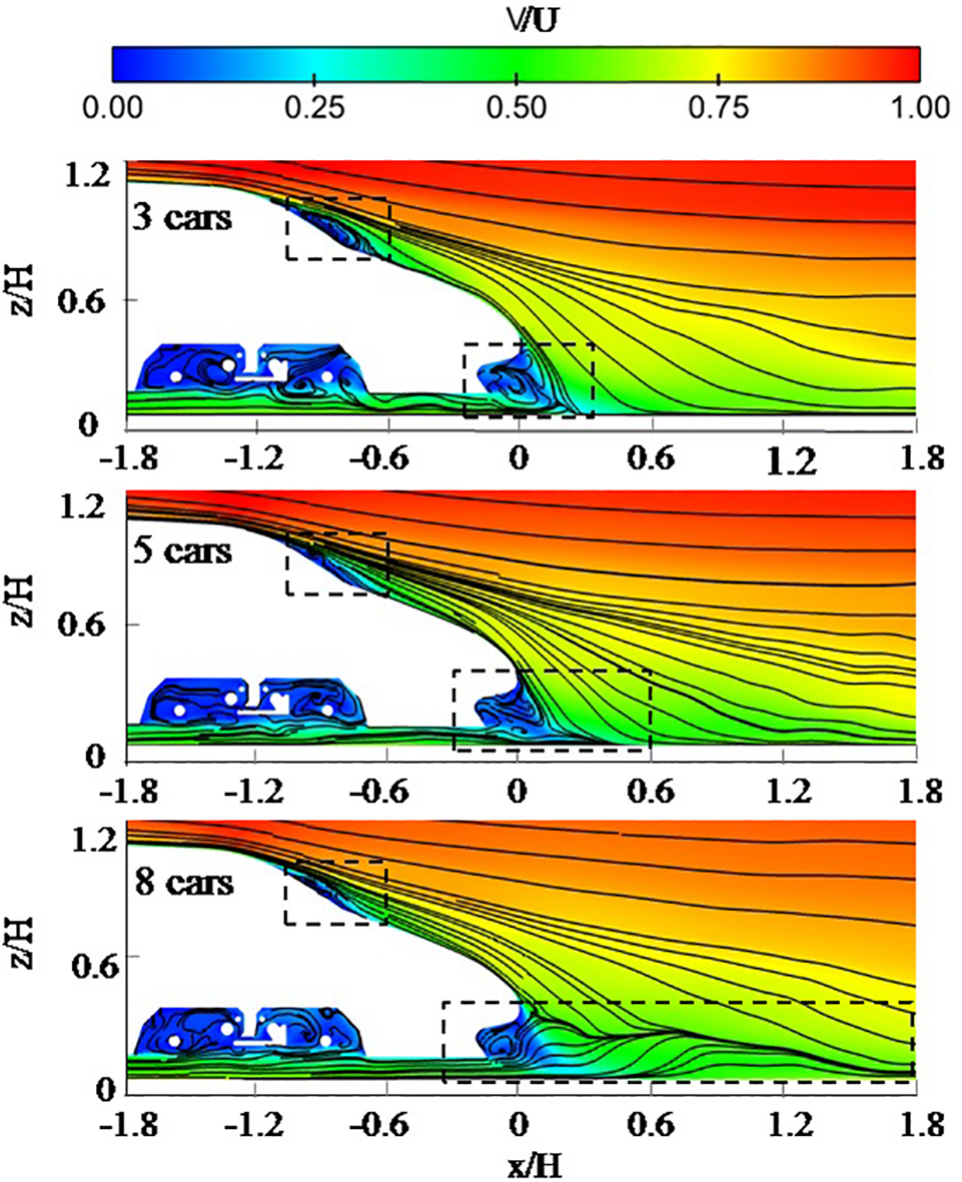
Velocity distribution and particle trace on a longitudinal plane.

Fig 8 shows the velocity distribution and particle trace on the longitudinal plane in 3 cars, 5 cars and 8 cars. Fig 8 shows similar wake flows for trains with different lengths. The downwash flow from the roof of the train interacts with the up wash flow from the under body, forming 2 separation bubbles. One exists in the streamlined transition of the tail car, the other is between the nose and the cowcatcher of the tail car. And a reattachment region is observed behind the train. The separation bubble of the streamlined transition is smaller in longer trains. This could be because the energy dissipation increases as the boundary-layer thickness increases. Consequently, the turbulence energy of the boundary layer in the tail car belonging to the longer train is lower. The separation bubble between the nose and the cowcatcher grows larger and the reattachment length becomes longer with the longer train. The reattachment length is 0.3H in 3 cars, 0.45H in 5 cars, 1.7H in 8 cars.

**Fig 9:**
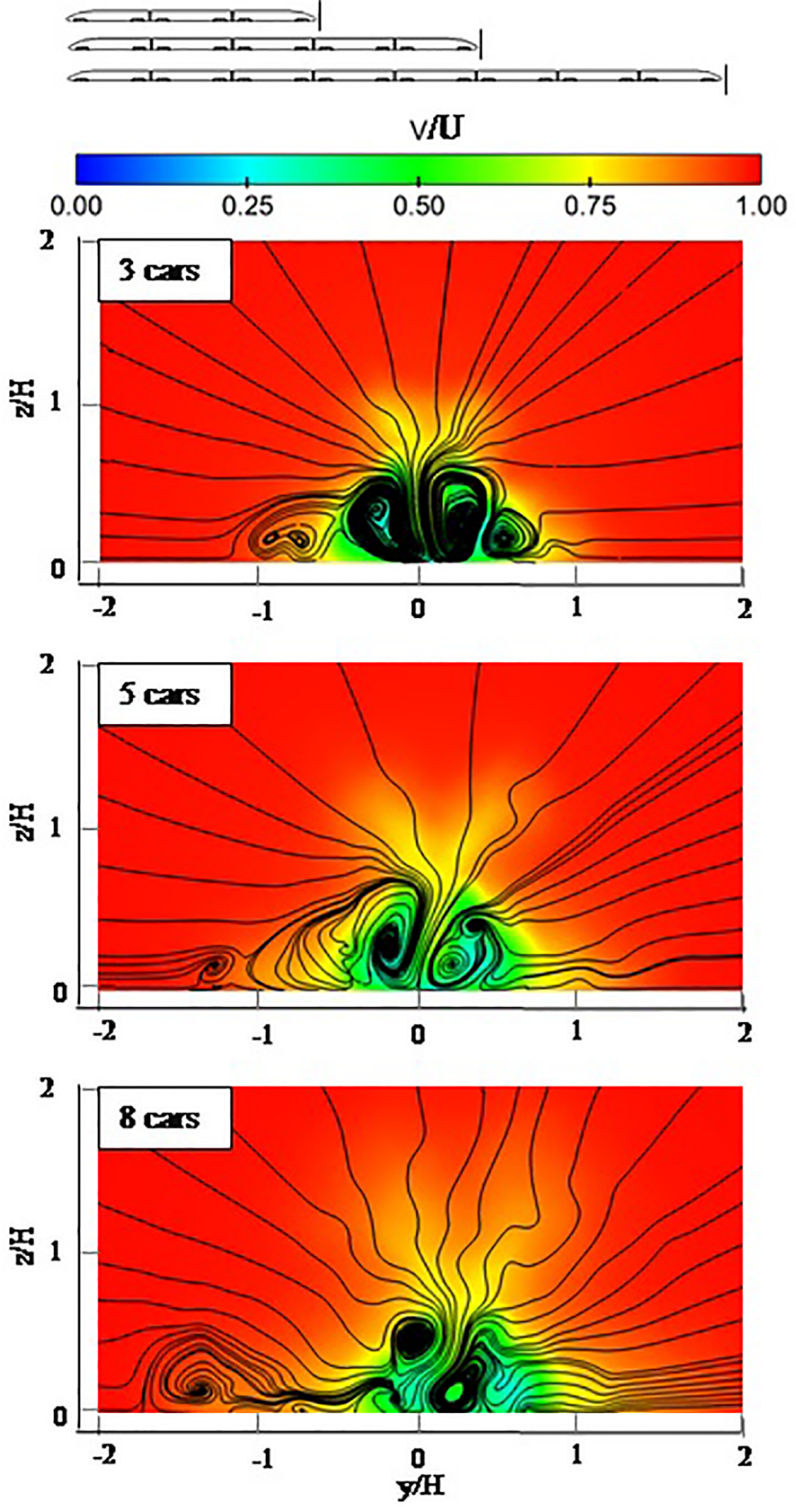
Velocity distribution and particle trace on a vertical plane at 0.3H from the nose of the tail(G-G shown in the Fig 6A).

Fig 9 shows the velocity distribution and particle trace on a vertical plane at 0.3H from the nose of the tail in 3 cars, 5 cars and 8 cars. Fig 9 shows similar wake flows for trains with different lengths. There is a pair of counter rotating vortices, and the two dominant vortices in the near wake of 3 cars are symmetric. However, the two dominant vortices in the near wake become less symmetric as the train’s length increases. Fig 9 also shows some small-scale vortices around the dominant vortices. Additionally, the distance between the small vortices and the dominant vortices grows larger with the train’s increasing length.

### Aerodynamic performance of trains with differing lengths

**Fig 10:**
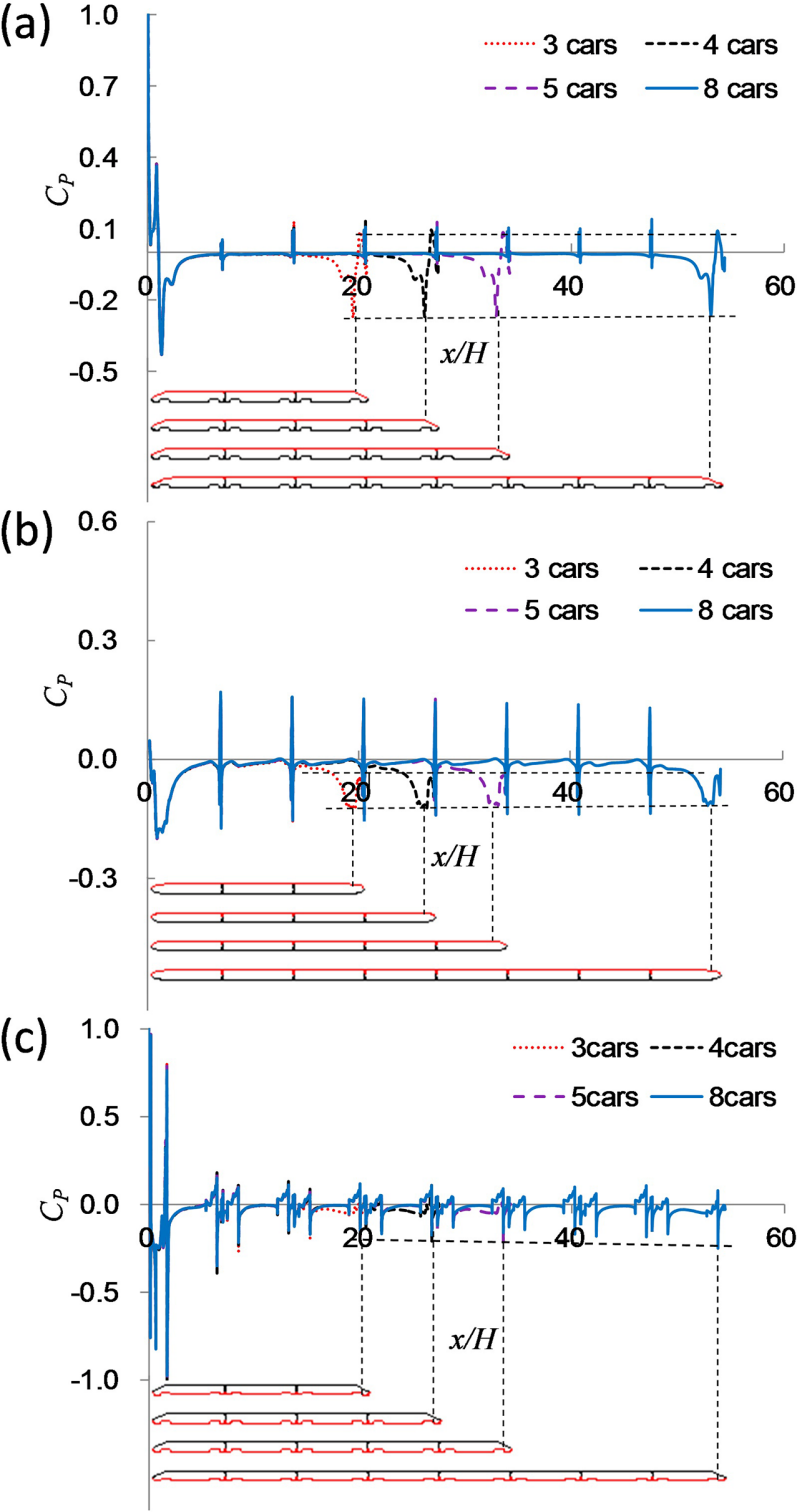
*C*_*P*_ along the length of the train: (a) *C*_*P*_ along the upper of the train; (b) *C*_*P*_ along the side of the train; (c) curve fitting between the negative peak of the tail car and the train length.

To analyze the characteristic of the surface pressure in trains with differing lengths, the pressure coefficient (*C*_*P*_) along the upper portion of the train, along the side of the train located at 0.5H and along the train’s underside (without bogies) are shown in Fig 10. The direction of train length is normalized using H.

Fig 10(A) shows large fluctuations in the streamlined head, the streamlined rear and the inter-carriage gaps. The rest is negative and closes to 0. The train length has little effect on *C*_*P*_ of the head car, but it has a great effect on *C*_*P*_ of the tail car. The negative peak of the tail car appears near the streamlined transition and it decreases with the increasing length of the train. The value is 0.3668 in 3 cars, 0.3561 in 8 cars, and *C*_*P*_ is reduced by 2.9%. This could be the reason for the increased dissipation of the energy in the boundary layer caused by the inter-carriage gaps, after which parts the separation strength decreases with the increasing length of the train.

Fig 10(B) shows large fluctuations in the streamlined head, the streamlined rear and the inter-carriage gaps. The train length has little effect on the train head, but has a significant effect on *C*_*P*_ of the tail car. The negative peak of the tail car appears near the streamlined transition and decreases obviously with the train’s increasing length. The value is 0.1037 in 3 cars, 0.0951 in 8 cars, and *C*_*P*_ is reduced by 8.3%. This trend is similar with the upper surface pressure, because the boundary layer development is dominant in both the upper and side areas of the train [12].

In Fig 10(C), there are large fluctuations in the streamlined head, the streamlined rear, the inter-carriage gaps and the bogie cabins. The train length has little effect on the train head, but has a great effect on *C*_*P*_ of the tail car. The negative peak of the tail car appears in the last bogie cabins and increases significantly with the train’s increasing length. The value is 0.2080 in 3 cars, 0.2490 in 8 cars, and *C*_*P*_ increases by 19.7%. The maximum velocity in the downstream portion of the train increases along the length of the train. The separation strength increases with the higher velocity. As a result, lower negative pressure in the streamlined transition of the tail car and the higher negative pressure in the last bogie cabin of the tail car lead to a lower pressure drag with longer trains.

According to the previous analysis, the boundary-layer thickness relates to the train length, and boundary-layer thickness is an important factor affecting frictional drag. As such, the friction drag coefficient(*C*_*Dτ*_) for trains with differing lengths is shown in Table 2. It can be seen that *C*_*Dτ*_ of each car in the same configuration depends on the location, and there is a decreasing trend along the length of the train. This is why the boundary-layer thickness becomes thicker with longer trains. As a result, the velocity gradient in the boundary layer shrinks, and the viscous shear stress of the train surface becomes smaller in longer trains. The sum of the viscous shear stress is the friction drag, so *C*_*Dτ*_ decreases from the head car to the tail car. The *C*_*Dτ*_ for the head car in trains with differing lengths varies from 0.0459 to 0.0462, and the biggest change is within 0.7%. The *C*_*Dτ*_ of the middle car varies from 0.0356 to 0.0418, but the biggest change in the same configuration occurs below 7.2%. The *C*_*Dτ*_ of the tail car varies from 0.0297 to 0.0324, and the biggest change occurs below 8.6%. A decreased trend occurs with the train’s increasing length.

**Table 2:** *C*_*Dτ*_ for trains with different lengths.

*C*_*Dτ*_	Head car	Middle car1	Middle car2	Middle car3	Middle car4	Middle car5	Middle car6	Tail car
3 cars	0.0459	0.0402	\	\	\	\	\	0.0323
4 cars	0.0460	0.0413	0.0380	\	\	\	\	0.0314
5 cars	0.0461	0.0415	0.0400	0.0360	\	\	\	0.0311
8 cars	0.0462	0.0418	0.0387	0.0386	0.0378	0.0360	0.0356	0.0297

To analyze the effect of the train length on the drag, the aerodynamic drag coefficient (*C*_*D*_) in 3 cars, 4 cars, 5 cars and 8 cars is shown in Table 3. It can be seen that *C*_*D*_ of the head car for trains with different lengths varies from 0.1437 to 0.1448, the biggest change is within 0.77%; *C*_*D*_ of the middle car varies from 0.0814 to 0.0827, and the biggest change is below 1.6%. There is no obvious trend about the train length to the drag and this is consistent with the result from the wind tunnel test [14]. *C*_*D*_ of the tail car is 0.1594 in 3 cars, 0.1535 in 8 cars, and *C*_*D*_ is reduced by 3.7%, which is the collective effect of the pressure drag and the friction drag.

**Table 3:** *C*_*D*_ for trains with different lengths.

*C*_*D*_	Head car	Middle car1	Middle car2	Middle car3	Middle car4	Middle car5	Middle car6	Tail car
3 cars	0.1439	0.0824	\	\	\	\	\	0.1594
4 cars	0.1437	0.0820	0.0823	\	\	\	\	0.1582
5 cars	0.1434	0.0819	0.0823	0.0817	\	\	\	0.1546
8 cars	0.1448	0.0814	0.0824	0.0827	0.0819	0.0817	0.0816	0.1535

## Conclusion

In this paper, the DDES was used to simulate trains of different lengths running in the open air. This work researched boundary layers, wake vortices, surface pressures, aerodynamic drag coefficients and friction drag coefficients. Certain conclusions can be drawn from the results obtained in this study.

The boundary-layer thickness around the train increases along the length of the train. Dominant vortices in the near wake become less symmetric, and the distance between small vortices and dominant vortices increases with longer trains. The separation bubble of the streamlined transition of the tail car is smaller, the separation bubble between the tail nose and the cowcatcher is larger and the reattachment region behind the train is likewise longer with longer trains.

The train length has little effect on the surface pressure of the head car, but it has a large effect on the surface pressure of the tail car. The negative peak of the upper and side of the tail car reduces with the train’s increasing length. The negative peak related to the underside of the tail car increases with the train’s length. The train length has a significant effect on friction drag coefficients. The friction drag coefficient of each car in a configuration decreases along the length of the train. The friction drag coefficient of the tail car also shows a decreased trend with a train’s increasing length. The same trend occurs in the drag coefficient of the tail car.
